# Hair Graying Regulators Beyond Hair Follicle

**DOI:** 10.3389/fphys.2022.839859

**Published:** 2022-02-24

**Authors:** Jing Chen, Yixin Zheng, Chen Hu, Xuexiao Jin, Xiaoping Chen, Ying Xiao, Chaochen Wang

**Affiliations:** ^1^Department of Breast Surgery, The Second Affiliated Hospital, Zhejiang University School of Medicine, Hangzhou, China; ^2^Zhejiang University – University of Edinburgh Institute, Zhejiang University, Haining, China; ^3^School of Medicine, Zhejiang University, Hangzhou, China; ^4^Institute of Immunology and Department of Rheumatology, Sir Run Run Shaw Hospital, Zhejiang University School of Medicine, Hangzhou, China; ^5^Central Lab of Biomedical Research Center, Sir Run Run Shaw Hospital, Zhejiang University School of Medicine, Hangzhou, China

**Keywords:** hair graying, melanocyte stem cells, nerves, adipocytes, immune cells

## Abstract

Hair graying is an interesting physiological alteration associated with aging and certain diseases. The occurrence is due to depigmentation of the hair caused by depletion and dysfunction of melanocyte stem cells (MeSCs). However, what causes the depletion and dysfunction of MeSCs remains unclear. MeSCs reside in the hair follicle bulge which provides the appropriate niche for the homeostasis of various stem cells within hair follicle including MeSCs. In addition to local signaling from the cells composed of hair follicle, emerging evidences have shown that nerves, adipocytes and immune cells outside of hair follicle *per se* also play important roles in the regulation of MeSCs. Here, we review the recent studies on different cells in the MeSCs microenvironment beyond the hair follicle *per se*, discuss their function in regulating hair graying and potentially novel treatments of hair graying.

## Introduction

Hair graying is one of the representative signs of aging. It has been considered to be triggered by a decreased number of follicular melanocyte stem cells (MeSCs; Nishimura et al., 2005; Iida et al., 2020) or dysfunction of MeSCs such as decreased oxidation resistance capacity with aging (Shi et al., 2014). Hair graying in people younger than 30 years old is termed premature canities which can cause an adverse effect on the self-esteem (Tobin and Paus, 2001). Premature hair graying is also associated with many diseases, such as cancer, pernicious anemia, and hyper/hypo-thyroidism. Certain medications including Chloroquine, Tamoxifen, and Pazopanib, can also induce hair graying (Ricci et al., 2016; Acer et al., 2020). Regardless of the advances in the study of hair graying, the mechanisms underlying depigmentation are poorly understood.

**Figure 1:**
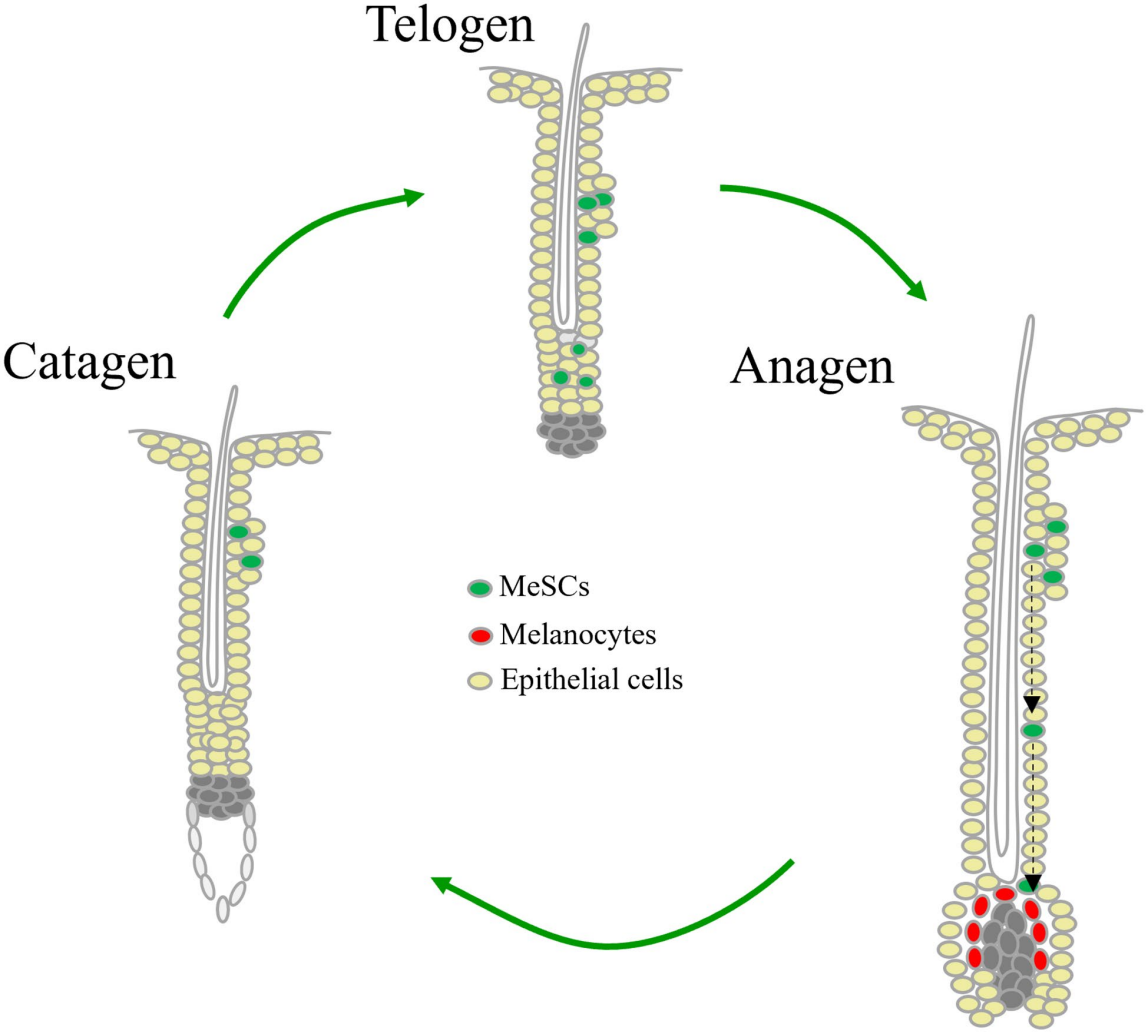
Cyclical regeneration of melanocyte stem cells during hair cycling.

MeSCs, derived from neuronal crest, mainly locate within the hair bulge area in hair follicle. The proliferation and differentiation of MeSCs are in parallel with hair follicle cycle (Figure 1). Micro-ophthalmia-associated transcription factor (MITF) is a master regulator that promotes melanocytic development and melanogenesis by upregulating many melanogenesis-related genes, including tyrosinase, tyrosinase-related protein 1 and dopachrome tautomerase (DCT; Hou and Pavan, 2008). Expression of MITF can be stimulated by PAX3, a transcription factor which also directly represses DCT expression by competing with MITF maintaining MeSCs in an undifferentiated state (Lang et al., 2005). This homeostasis can be disrupted by WNT/β-catenin activation (Lang et al., 2005). At the onset of pigmented hair regeneration in anagen phase, MeSCs receive elevated signaling such as WNT signaling, endothelins, α-MSH, and KITL from surrounding hair follicle stem cells (HFSCs) and the inferior dermal papilla, directing MeSCs proliferate and migrate to hair bulb, and differentiate into melanocytes to synthesize melanin which is transported to adjacent precortical keratinocytes of hair shaft (Slominski et al., 2005; Li and Hou, 2018; Lim et al., 2019; Qiu et al., 2019). Toward the end of anagen, melanogenesis is shut down, melanocytes undergo apoptosis in the following catagen and telogen, and MeSCs surviving from hair cycles become quiescent by factors like TGF-β, Notch ligands JAG and DLL1, and WNT inhibitors SFRP and DKK3 secreted by HFSCs (Qiu et al., 2019).

Cross-talks between stem cells and their surrounding microenvironments are critical for the maintenance and self-renewal of stem cells. The role of HFSCs and dermal papilla cells have been intensively investigated in MeSCs niche for decades. However, other types of cells including neural cells, adipocytes, and immune cells are also important components of the hair bulge niche and can also affect MeSCs, especially in some pathological conditions. Here, we reviewed recent studies on the mechanisms of hair graying induced by cells outside hair follicles and try to offer some new insights for clinical treatment of hair graying.

## Neural Microenvironment

Stress has been considered as a major risk factor for hair graying for decades. Several cases of rapidly graying after stress have been reported in both human and mouse (Ephraim, 1959; Peters et al., 2017; Shen et al., 2020). The autonomic nervous system (ANS) and the hypothalamo-pituitary-adrenocortical axis are major effectors responding to stresses (Ulrich-Lai and Herman, 2009). ANS immediately responds to stresses through sympathetic or parasympathetic nerves. Hair follicles are innervated by several types of nerves including sympathetic nerves and sensory nerves, which enwrap the infundibulum, isthmus and bulge of hair follicles (Botchkarev et al., 1997; Crawford and Caterina, 2020). Sensory nerves form highly arranged lanceolate nerve endings at the isthmus and bulge region, which are longitudinally and circularly oriented, respectively. In contrast, sympathetic nerve fibers enwrap and penetrate the arrector pili muscle and extend to the hair follicle bulge and the hair germ (Botchkarev et al., 1999; Zhang et al., 2020). The plasticity of hair follicle innervation of both sensory and sympathetic nerves is hair cycle-dependent, especially a significant transient increase in longitudinal fiber number is at the early stage of anagen (Botchkarev et al., 1997). Given nerves are in close contact with hair bulges where MeSCs reside (Zhang et al., 2020), signals from nerves should also have direct impact not only on hair follicles but also on MeSCs. Additionally, it has been shown that hair graying was retarded in two patients with cervical or lumbar sympathectomy (Lerner, 1966; Ortonne et al., 1982), indicating that sympathetic nerves might play a role in hair pigmentation.

Recently, Hsu and her colleagues found a solid link between sympathetic nerves and hair graying, revealing its underlying mechanism in mouse models (Zhang et al., 2020). They showed that noradrenaline, released by sympathetic nerves that were hyper-activated by acute stresses, forces MeSCs but not mature melanocytes to enter a rapid but abnormally proliferative state. These MeSCs differentiate and migrate out of the hair bulge, resulting in a permanent, irreversible loss of MeSCs, which ultimately leads to hair graying. However, MeSCs loss can be prevented if the proliferation of MeSCs was suppressed early in the stress response through inducing the expression of P27 which is an inhibitor of cyclin-dependent kinase. These preserved MeSCs with normal morphology and functionality can recolor newly regenerated hairs in next hair cycle. Consistently, a more recent study in humans showed that hair graying and hair recoloring can occur in parallel with stress exposures in young individuals of 9–39 years old (Rosenberg et al., 2021). Hair graying and recoloring can be observed within a single hair shaft in humans but not in mice. A possible reason is that humans have a much longer anagen period of hair follicles. On the other hand, Hsu et al. also have demonstrated that stress-induced corticosterone and noradrenaline released by adrenal glands and stress-induced immune attacks have no effect on hair pigmentation (Zhang et al., 2020). Together, these results indicate that signals from nerves surrounding MeSCs niche (hair bulge) play an essential role in regulating MeSCs’ functions with exposure of stresses.

Sonic hedgehog (SHH) signaling, a major regulator of cell differentiation and proliferation, has been reported to stimulate the proliferation of melanocytes that are isolated from human epidermis *in vitro*. Over-activation of SHH pathway results in proliferation and survival of melanomas by regulating downstream GLI1 and the RAS–MEK/AKT pathways (Stecca et al., 2007). Brownell et al. have demonstrated that sensory neurons projected from dorsal root ganglions (DRGs) around hair follicle bulge can secret SHH (Brownell et al., 2011), suggesting that nerve-derived SHH may also participate in pigmentation. Moreover, single-cell analysis of mouse DRG reveals that *Tgfb, Wnt*, and *Kitl*, which are essential factors for the maintenance of melanocytes or MeSCs, are detectable in DRGs (Usoskin et al., 2015). Further studies are required to investigate whether nerves projected to hair follicles secrete these factors and whether these factors modulate melanogenesis.

Peripheral nerve axons are supported by variable glial cells including Schwann cells (SCs). Both longitudinal and circular nerve fibers at the level of hair follicle bulge are accompanied by SCs. A previous study shows that melanocytes and SCs share the same progenitor called Schwann cell precursors (Adameyko et al., 2009). When peripheral nerve injury occurs, SCs dedifferentiate and transdifferentiate into melanocytes by activating MAP kinase pathway through inhibiting neurofibromin, which results in consequent pigmentation of skin dermis (Van Raamsdonk and Deo, 2013). These results provide a new strategy to treat hair graying in clinic: transplanting SCs-derived melanocytes or promoting the transdifferentiation of SCs around hair follicles. However, whether SCs could modulate hair follicle melanocytes needs to be further studied.

## Adipocytes

There are abundant adipocytes in skin dermis, referred as dermal white adipose tissue (dWAT; Kruglikov and Scherer, 2016). The dWAT exists below the reticular dermis as well as surrounding hair follicles, and is separated from subcutaneous WAT in rodents by the panniculus carnosus that is absent in many mammals including human beings. dWAT is histologically and functionally distinguished from subcutaneous WAT (Driskell et al., 2014). dWAT has been demonstrated to be involved in several physiological and pathological processes, including immune response, wound healing and scarring, and hair follicle growth (Alexander et al., 2015; Kruglikov and Scherer, 2016). dWAT changes along with hair cycles in both rodents and humans (Guerrero-Juarez and Plikus, 2018). In anagen, when melanin synthesis is activated, adipocytes in dWAT show larger size, increased cell number, and higher lipid metabolic activity, especially at the hair bulge (Nicu et al., 2021b). Growth factors derived from adipocytes of dWAT greatly affect hair follicle cycles (Hesslein et al., 2009; Festa et al., 2011; Guerrero-Juarez and Plikus, 2018). dWAT is a key component of hair follicle niche, and previous studies have shown that obesity and higher BMI are correlated with premature hair graying (Kocaman et al., 2012; Shin et al., 2015), which gives rise to a hypothesis that adipocytes could affect hair pigmentation in certain ways.

Recently, Nicu et al., have found that perifollicular dWAT significantly stimulates hair pigmentation by hepatocyte growth factor (HGF) but inhibits melanogenesis by adiponectin which is an adipocytokine. HGF, secreted mainly by adipocyte progenitors and pericytes in anagen perifollicular dWAT, stimulates WNT/β-catenin activity in hair matrix by inhibiting WNT antagonist SFRP1 as well as upregulating *WNT10B* that promotes melanocytes maturation and pigmentation (Ye et al., 2013; Nicu et al., 2021b). Adiponectin oligomers, however, downregulates *KITL*, *TYRP1* and *WNT10B*, and inhibits the expression of the HGF receptor c-Met within hair matrix (Nicu et al., 2021a). Adiponectin also circulates as a globular fragment, which is evidenced to stimulate melanogenesis by upregulating MITF through MAPK signaling pathway (Kim et al., 2018b). Therefore, the ratio of adiponectin oligomer to globular adiponectin, or the ratio of HGF to adiponectin is important in sustaining melanin synthesis.

On the contrary, adipocytes in dWAT isolated from human abdomen produce abundant TGF-β1 and interleukin-6 (IL-6) and suppress pigmentation in skin by inhibiting several key melanogenic enzymes such as tyrosinase (Kim et al., 2014; Klar et al., 2017). These results suggest that dWATs in different tissues are likely to regulate melanogenesis in different ways. TGF-β signaling is demonstrated to downregulate MITF and its downstream melanogenic genes, and promotes MeSCs re-entering into the quiescent state at the end of anagen in mice (Nishimura et al., 2010), which raises the possibility that perifollicular dWAT in human might also secret TGF-β1 to maintain MeSCs during the anagen-catagen transition of hair follicles. Additionally, a previous study has shown that HGF could inhibit TGF-β1 by up-regulating Smad transcriptional corepressor TG-interacting factor (TGIF; Dai and Liu, 2004). However, only few research about the relationship between dWATs and MeSCs in hair follicles has been performed, and all studies in human by far were conducted *in vitro*; therefore, further investigations are required.

## Immune Cells

Immune microenvironment plays an important role in regulating skin homeostasis, wound healing, as well as hair cycles. Similar to nerves and dWATs, types and numbers of immune cells also change along with hair cycles (Wang and Higgins, 2020). FGF-5 released by macrophages could induce catagen in both rats and humans (Suzuki et al., 1998; Higgins et al., 2014). The numbers of macrophages increase during telogen progression but decrease in period between late telogen and early anagen in mice (Castellana et al., 2014). Mast cells and Treg cells accumulate around the hair bulge area during the telogen–anagen transition, promoting anagen entry and immune privileges that prevents autoimmune attack of the shifting antigens in the growing hair follicles (Bertolini et al., 2020; Wang and Higgins, 2020). Melanocyte- and melanogenesis-associated epitopes are considered as autoantigens for T cells and significantly induce higher responses of cytotoxic CD8+ T cells to attack hair follicle cells resulting in alopecia areata (Wang et al., 2016; Bertolini et al., 2020). Hypopigmented or gray hair can be also observed in alopecia areata patients (Asz-Sigall et al., 2019), suggesting the possibility that immune cells participate in the development of hair graying.

Programmed death-ligand 1 (PD-L1) suppresses T cells by binding to PD-1 on T cells. PD-L1 is highly presented in melanocytes in melanoma and plays an important role in the establishment of immune privilege (Taube et al., 2012; Willemsen et al., 2020). Dimitriou et al. have reported a case of a female patient with stage IIID melanoma showed hair depigmentation after being treated by anti-PD1 antibody, BRAF and MEK inhibitors (Dimitriou et al., 2020), indicating a reduced peripheral tolerance to melanocytic self-antigen. In Pmel-1 vitiligo mice presenting gray hair, PD-L1 treatment, however, could significantly reverse and suppress depigmentation development in adults by recruiting Treg cells to maintain the normal immune privilege for melanocytes and to repress the abundance of melanocyte-reactive T cells (Miao et al., 2018; Chen et al., 2021). In addition, using human tyrosinase epitope-reactive T-cell receptor cloned from tumor-infiltrating T lymphocytes of a metastatic melanoma patient, Mehrotra et al. established transgenic mice and found that these mice spontaneously developed severe hair depigmentation (Mehrotra et al., 2012).Together, these data suggest that melanocyte-targeting T cells that are enriched in the skin under certain pathological conditions could induce death and dysfunction of melanocytes, leading to subsequent hair graying. However, how different T cells change and co-ordinate under pathological conditions and how to maintain the homeostasis of different T cells in the healthy body remain to be clarified.

Additionally, several studies have shown that melanocytes can function as antigen presenting cells (APCs) and secrete inflammatory cytokines. Similar to canonical APCs, melanocytes treated with interferon-γ (IFN-γ) express MHC II and secret cytokines like IL-1α, IL-1β, IL-8, and TGF-β1 (Zachariae et al., 1991; Swope et al., 1994; Hedley et al., 1998; Le Poole and Boyce, 1999). Innate immune receptor Toll-like receptors (TLRs) are also detected in melanocytes, and agonists of TLR2/3/4/7/9 can stimulate the production and secretion of cytokines and chemokines including IL-6, IL-8 and CCL2/3/5 through regulating nuclear translocation of NFkB p65 and phosphorylation of IkBα (Yu et al., 2009; Koike and Yamasaki, 2020), indicating that melanocytes play a role in enhancing the innate immune response. On the other hand, these TLRs are proved to either promote or inhibit melanogenesis in melanocytes (Koike and Yamasaki, 2020). In addition, TLR3 also participates in the release, transfer, uptake, and degradation of melanin (Koike et al., 2019). However, it is worth to mention that all the expression and function of TLRs have been investigated in human epidermal melanocytes rather than hair follicle MeSCs and melanocytes. Furthermore, a recent study has found that MITF can also negatively regulate the expression of innate immune genes in melanocytes *in vitro*. Haploinsufficiency of *Mitf* in mice leads to an elevated and sustained IFN-regulated genes (IFN signature) expression in MeSCs that exacerbates MeSC differentiation and hair graying, highlighting a negative effect of intrinsic innate immune activation on MeSC and melanocyte (Harris et al., 2018). The fact that MeSCs or melanocytes can function as immune cells provides new insights into the mechanism of hair graying.

## Conclusion and Perspectives

**Figure 2:**
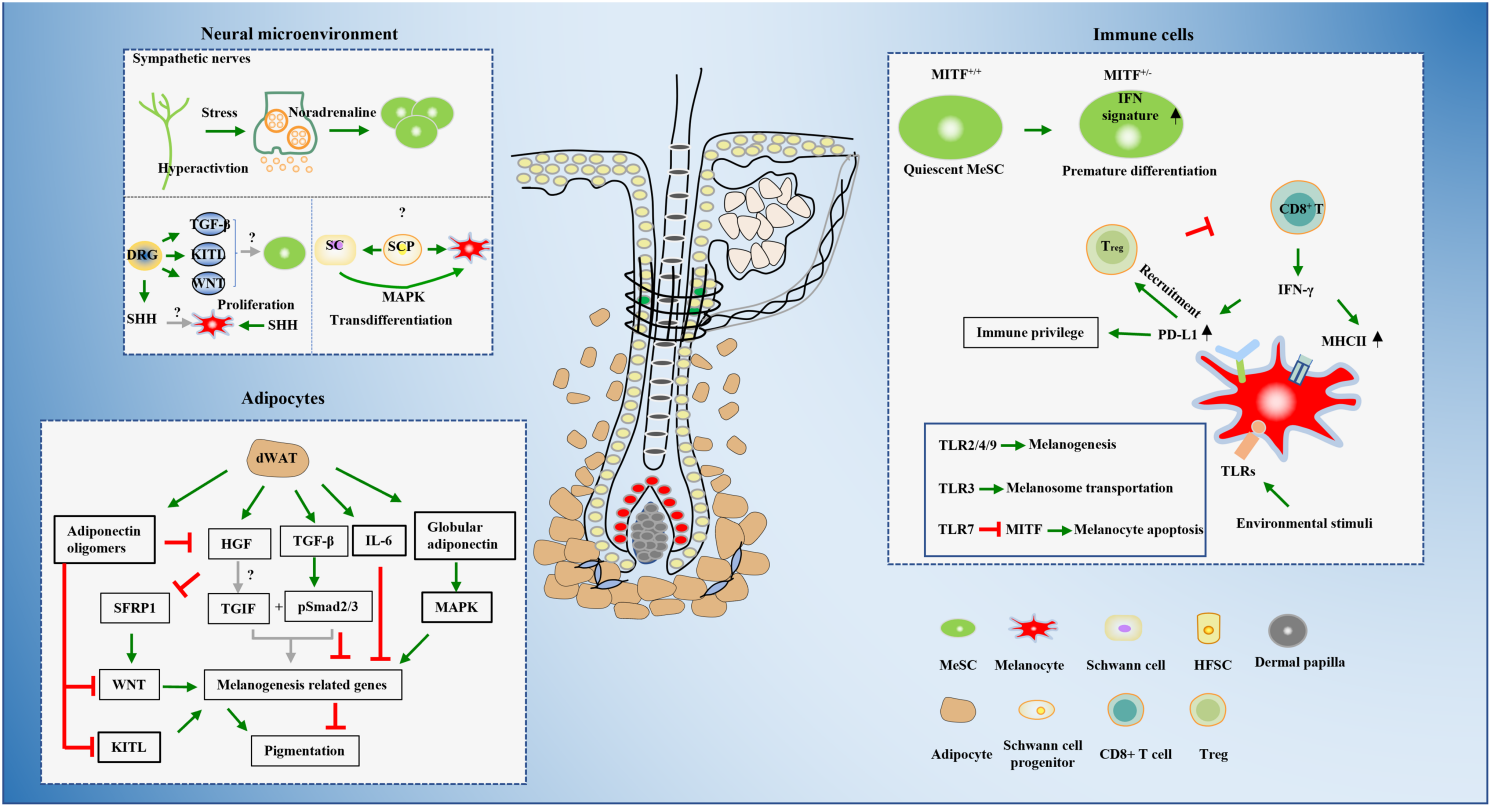
Summaries of the cellular microenvironments that have been evidenced or are likely to be involved in hair graying.

Pigmentation is a multi-step process which involves multiple types of cells, therefore, disruption of any step can lead to hair graying in reversible or irreversible ways. Cells reside in sophisticated but well-organized cellular microenvironments that provide the basis for biological function. In addition to the intrinsic factors of MeSCs and melanocytes and their closely contacted hair follicle cells in hair follicle bulge, bulb, and dermal papillae cells, other types of cell, including nerves, adipocytes, and immune cells, are also emerging as functional components of the microenvironments of MeSCs and melanocytes (Figure 2).

In addition to the cell types mentioned above, fibroblasts and microvessels also make up a large portion of skin enwrapping hair follicles, which have been reported to greatly affect the pigmentation of epidermis. Fibroblasts secret a diversity of factors participate in modulating melanogenesis: KITL and KGF induce the proliferation and survival of melanocytes by MAPK signaling pathway, DKK1 and SFRP modulate pigmentation *via* WNT/β-catenin pathway (Wang et al., 2017). In pathological conditions, such as melasma and solar lentigo, factors stimulating melanogenesis are over expressed and secreted by fibroblasts resulting in hyperpigmentation (Wang et al., 2017). A very recent study showed that fibroblasts is the dominant cell type in the skin that responds to IFN-γ in vitiligo, which recruit and active CD8+ cytotoxic T cells to eliminate melanocytes (Xu et al., 2022). On the other hand, endothelial cells co-cultured with melanocytes produce endothelin-1 activate melanin synthesis by binding to the endothelin receptor B, which explains the close relationship between pigmentation and the vasculature in melasma (Regazzetti et al., 2015). Conditioned medium obtained from endothelial cells, however, are demonstrated to inhibit pigmentation by secreting TGF-β and clusterin (Park et al., 2016; Kim et al., 2018a). Further studies are required to understand whether fibroblasts and endothelial cells are also involved in regulating hair follicular MeSCs.

Although hair graying is usually not a medical problem, it has afflicted many people because of the esthetic problem. While hair graying caused by vitamin B12 deficiency or hypothyroidism can be reversed by vitamin supplement or hormone replacement, respectively. Most individuals with hair graying must rely on colorants to recolor their hairs (Kumar et al., 2018). However, commercial permanent colorants are toxic and could cause damage to the hair shaft, and people have to perform hair coloring repeatedly due to continuous growth of hair shaft, resulting in irritant dermatitis and hair loss in some cases (Trueb, 2006). Hence, new strategies targeting the biological processes of melanogenesis are needed. Although, all cells in the skin can communicate with each other directly or indirectly, the predominant cell types with aberrant secretome might be different in different diseases or aging individuals. Understanding the MeSCs and melanocyte niches will be helpful to advance the research of on melanogenesis and chose the priority cell type for clinical treatments for hair graying.

## Author Contributions

JC conceived the idea for the manuscript and drafted the manuscript with significant contribution from YZ, CH, XJ, and XC. YZ and JC designed the figures. YX and CW revised the manuscript. All authors contributed to the article and approved the submitted version.

## Funding

This work was supported by National Natural Science Foundation of China (31900620).

## Conflict of Interest

The authors declare that the research was conducted in the absence of any commercial or financial relationships that could be construed as a potential conflict of interest.

## Publisher’s Note

All claims expressed in this article are solely those of the authors and do not necessarily represent those of their affiliated organizations, or those of the publisher, the editors and the reviewers. Any product that may be evaluated in this article, or claim that may be made by its manufacturer, is not guaranteed or endorsed by the publisher.
